# Performance of ChatGPT-4o, Claude 3 Opus, and DeepSeek-R1 in BI-RADS Category 4 Classification and Malignancy Prediction From Mammography Reports: Retrospective Diagnostic Study

**DOI:** 10.2196/80182

**Published:** 2025-12-25

**Authors:** Xingwei Dai, Man Ke, Dixing Xie, Mengting Mei, Si Wei, Yi Dai, Ronghua Yan

**Affiliations:** 1 Peking University Shenzhen Hospital Shenzhen China; 2 Southern University of Science and Technology Shenzhen China

**Keywords:** large language models, BI-RADS, breast, mammography, ChatGPT-4o, Claude 3 Opus, DeepSeek-R1, Breast Imaging Reporting and Data System

## Abstract

**Background:**

Mammography is a key imaging modality for breast cancer screening and diagnosis, with the Breast Imaging Reporting and Data System (BI-RADS) providing standardized risk stratification. However, BI-RADS category 4 lesions pose a diagnostic challenge due to their wide malignancy probability range and substantial overlap between benign and malignant findings. Moreover, current interpretations rely heavily on radiologists’ expertise, leading to variability and potential diagnostic errors. Recent advances in large language models (LLMs), such as ChatGPT-4o, Claude 3 Opus, and DeepSeek-R1, offer new possibilities for automated medical report interpretation.

**Objective:**

This study aims to explore the feasibility of LLMs in evaluating the benign or malignant subcategories of BI-RADS category 4 lesions based on free-text mammography reports.

**Methods:**

This retrospective, single-center study included 307 patients (mean age 47.25, 11.39 years) with BI-RADS category 4 mammography reports between May 2021 and March 2024. Three LLMs (ChatGPT-4o, Claude 3 Opus, and DeepSeek-R1) classified BI-RADS 4 subcategories from the reports’ text only, whereas radiologists based their classifications on image review. Pathology served as the reference standard, and the reproducibility of LLMs’ predictions was assessed. The diagnostic performance of radiologists and LLMs was compared, and the internal reasoning behind LLMs’ misclassifications was analyzed.

**Results:**

ChatGPT-4o demonstrated higher reproducibility than DeepSeek-R1 and Claude 3 Opus (Fleiss κ 0.850 vs 0.824 and 0.732, respectively). Although the overall accuracy of LLMs was lower than that of radiologists (senior: 74.5%; junior: 72.0%; DeepSeek-R1: 63.5%; ChatGPT-4o: 62.4%; Claude 3 Opus: 60.8%), their sensitivity was higher (senior: 80.7%; junior: 68.0%; DeepSeek-R1: 84.0%; ChatGPT-4o: 84.7%; Claude 3 Opus: 92.7%), while specificity remained lower (senior: 68.3%; junior: 76.1%; DeepSeek-R1: 43.0%; ChatGPT-4o: 40.1%; Claude 3 Opus: 28.9%). DeepSeek-R1 achieved the best prediction accuracy among LLMs with an area under the receiver operating characteristic curve of 0.64 (95% CI 0.57-0.70), followed by ChatGPT-4o (0.62, 95% CI 0.56-0.69) and Claude 3 Opus (0.61, 95% CI 0.54-0.67). By comparison, junior and senior radiologists achieved higher area under the receiver operating characteristic curves of 0.72 (95% CI 0.66-0.78) and 0.75 (95% CI 0.69-0.80), respectively. DeLong testing confirmed that all three LLMs performed significantly worse than both junior and senior radiologists (all *P*<.05), and no significant difference was observed between the two radiologist groups (*P*=.55). At the subcategory level, ChatGPT-4o yielded an overall *F*_1_-score of 47.6%, DeepSeek-R1 achieved 45.6%, and Claude 3 Opus achieved 36.2%.

**Conclusions:**

LLMs are feasible for distinguishing between benign and malignant lesions in BI-RADS category 4, with good stability and high sensitivity, but relatively insufficient specificity. They show potential in screening and may assist radiologists in reducing missed diagnoses.

## Introduction

Breast cancer remains one of the leading causes of cancer-related death among women worldwide. Accurate and efficient diagnosis, along with appropriate clinical management, is critical for improving patient outcomes [1]. The Breast Imaging Reporting and Data System (BI-RADS) provides a standardized framework for assessing the risk of breast lesions. BI-RADS category 4 carries a wide range of malignancy probabilities, from 2% to 95%, with considerable overlap between benign and malignant findings [2]. Consequently, the precise classification and characterization of BI-RADS category 4 lesions remain challenging. Clinical guidelines recommend image-guided biopsy as the next step for BI-RADS 4 lesions, which inevitably leads to unnecessary invasive procedures in patients with benign conditions, increasing both economic and psychological burdens. Moreover, current diagnostic practices rely heavily on radiologists’ expertise. The subjectivity in interpretation and potential diagnostic errors may compromise the accuracy of category 4 assessments and subsequently influence patient management and treatment decisions [3].

In recent years, the rapid development of artificial intelligence (AI) has had a profound impact on the field of medical imaging, particularly through the rise of large language models (LLMs), such as the generative pretrained transformer (GPT), which possess outstanding capabilities in understanding human language [4]. ChatGPT, developed by OpenAI, is recognized for its sophisticated natural language processing abilities, leveraging a large-scale neural network trained on diverse textual data to generate coherent and contextually relevant outputs [5,6]. Similarly, Claude 3, created by the AI startup Anthropic, is designed to provide advanced cognitive performance and intelligent task handling. DeepSeek, a more recent entrant in the AI landscape, has attracted considerable attention for its efficient and open-source LLM architecture. It demonstrates forward-thinking design based on a native sparse attention mechanism that significantly improves traditional AI models in both training and inference efficiency, particularly enhancing long-context reasoning while maintaining performance and reducing pretraining costs [7,8]. Notably, DeepSeek is developed by a Chinese team and has been specially optimized for Chinese-language tasks, and multiple studies have already demonstrated its applicability in various medical domains [9-11].

Recent studies have highlighted the promising potential of LLMs in clinical applications, particularly in supporting decision-making processes and improving workflow efficiency across various medical specialties [12-14]. However, most research has primarily focused on converting clinical free text into structured formats, such as standardized summaries or classification labels [15-18]. In parallel, some studies have assessed the diagnostic reasoning or decision support capabilities of LLMs using free-text clinical inputs in controlled scenarios, such as imaging-based risk stratification and patient case assessments [19-23]. To date, only a few studies [24-27] have begun to explore the feasibility of using LLMs to directly generate diagnoses and clinical recommendations from free-text reports, such as those related to thyroid and musculoskeletal disorders. Preliminary investigations have suggested that LLMs have broad potential in assisting physicians with BI-RADS classification [28-30], but these studies have addressed the full spectrum of BI-RADS categories. Currently, no research has specifically focused on the particularly challenging BI-RADS category 4, where there is significant overlap between benign and malignant lesions. To our knowledge, no published studies have examined the use of LLMs to assist in the fine-grained subcategorization within BI-RADS category 4.

This study aims to evaluate the feasibility of using three LLMs (ChatGPT-4o, Claude 3 Opus, and DeepSeek-R1) to predict the specific subcategories of BI-RADS category 4 lesions based on free-text mammography reports and to further analyze the diagnostic reasoning behind their outputs.

## Methods

### Patients and Data

This study retrospectively collected mammography reports from patients who underwent breast cancer screening or diagnostic evaluation between May 2021 and March 2024. All reports were generated by breast radiologists certified by the institutional board. The reports were written and categorized in free-text format in Chinese. Each report was initially drafted by a junior radiologist (≤5 years of experience) based on imaging findings and subsequently reviewed by a senior radiologist (≥10 years of experience). The final version of each radiology report used as input to the LLMs was based on the senior radiologist’s revision, as senior radiologists had the authority to modify initial drafts during review. The final report explicitly assigned BI-RADS category 4 subcategories (4A, 4B, or 4C). The inclusion criteria were as follows: lesions classified as BI-RADS category 4 by the senior radiologist; patients who underwent surgical excision or core needle biopsy within 1 month after mammography and had a definitive pathological diagnosis; and reports containing complete imaging descriptions, including BI-RADS descriptors, impressions, and the final BI-RADS category assigned by the radiologist. The exclusion criteria included (1) a prior diagnosis of breast cancer or a history of breast surgery, radiotherapy, or chemotherapy and (2) follow-up cases after treatment.

### Ethical Considerations

The text processed by the LLMs is strictly confined to personal history reports. These reports were stripped of any information that could lead to patient identification, ensuring confidentiality and anonymity. The model’s interpretation of the texts focuses solely on identifying and structuring data relevant to the study without compromising individual privacy.

The study’s design and methodology were previously communicated to and reviewed by the hospital’s ethics committee. The research received the necessary approval, confirming that it adheres to the ethical standards required for patient data research. The study was granted an exemption from requiring informed consent due to its exclusive use of nonidentifiable data. Formal approval was obtained on June 9, 2025, under the reference number Peking University Shenzhen Hospital (Research) (2025) No.126.

### LLMs and Prompt Design

Three LLMs were used in this study: ChatGPT-4o (version May 24, 2024), Claude 3 Opus (version March 4, 2024), and DeepSeek-R1 (version January 20, 2025). All models were called between July 1, 2024, and March 15, 2025, with temperature set to zero and the default max-tokens setting. Each model responded to prompts in a single round.

Each model was instructed to act as an experienced breast radiology expert, providing specific BI-RADS classifications based on the input free-text reports. The following standardized prompt was used:

Assume you are a radiologist. Based on the following mammography report, please predict the benign or malignant nature of each nodule and provide a specific BI-RADS classification. Only one BI-RADS category is allowed. If classified as category 4, please clearly specify whether it is 4A, 4B, or 4C, and explain your diagnostic reasoning.

This prompt was designed to evaluate the model’s ability to interpret detailed imaging descriptions, make accurate classifications, and articulate its diagnostic rationale. No fine-tuning was performed on any of the three models, and all prompts were delivered in Chinese. The full Chinese prompt and example outputs from LLMs are provided in Multimedia Appendix 1, and a summary of prompt versions tested during pilot runs and their observed output limitations is presented in Multimedia Appendix 2.

### Workflow

**Figure 1:**
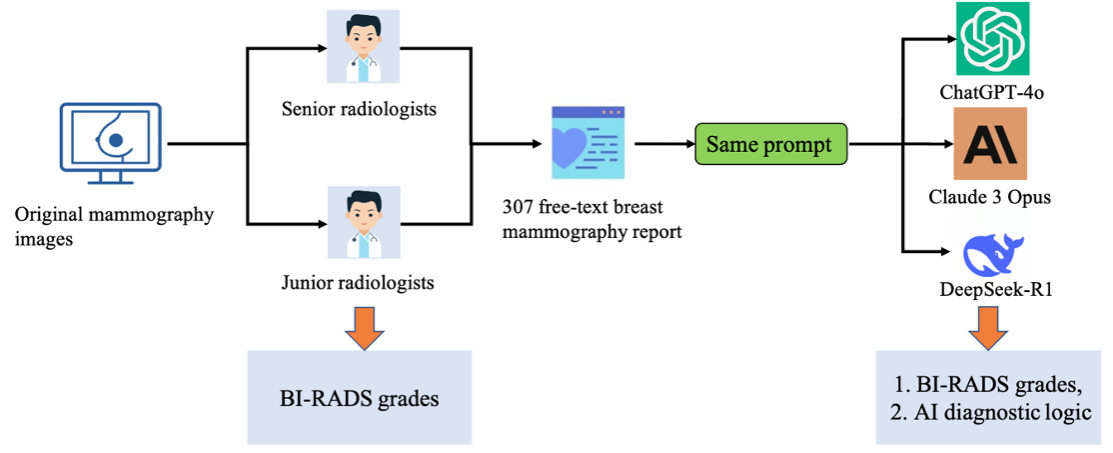
Schematic workflow of how large language models process free-text mammography reports and evaluate BI-RADS classifications. AI: artificial intelligence; BI-RADS: Breast Imaging Reporting and Data System.

The workflow of this study is illustrated in Figure 1. In this study, junior and senior radiologists interpreted the original mammography images to assign BI-RADS subcategories, whereas the LLMs analyzed only the deidentified free-text reports without access to imaging data. All mammography reports were deidentified before model input by removing patient-identifiable information and BI-RADS conclusions to ensure privacy and prevent label leakage. No further structural normalization or terminology unification was performed to preserve the natural language variability present in clinical practice [31]. Each report was analyzed by ChatGPT-4o, Claude 3 Opus, and DeepSeek-R1 using an open-ended prompt. To ensure consistency and reliability, each model was queried three times with the same report. All BI-RADS classification results from LLM outputs were manually extracted by two independent researchers. In cases of disagreement, a third researcher adjudicated the final decision to ensure accuracy and consistency. For each report, the most frequently occurring classification among the three responses was selected as the final result. If the three outputs differed without a clear majority, the case was deemed invalid and excluded from further analysis. This approach was designed to minimize variability and enhance the reliability of LLM-generated outputs. Additionally, we compared the consistency of BI-RADS classifications between LLMs and radiologists, as well as between junior and senior radiologists. To further explore the misclassification mechanisms of the LLMs, we manually reviewed the prediction rationales associated with all misclassified cases. Key imaging features, such as lesion size, calcification, shape, and margin characteristics, were extracted from the explanatory content generated by the LLMs and categorized in reference to the BI-RADS classification criteria. Feature extraction and interpretation were performed independently by two experienced radiologists. Any discrepancies were resolved through consensus discussions to ensure analytical rigor and objectivity.

### Statistical Analysis

Categorical variables were presented as frequencies (percentages), and continuous variables were expressed as means (SDs). In this study, lesions classified as BI-RADS 4A or below were considered benign, while those classified as 4B or above were considered malignant, in line with prior studies that have used this binary grouping to reflect increasing malignancy risk across subcategories [32,33]. Pathological diagnosis served as the reference standard only for binary classification (benign vs malignant), and a senior radiologist’s subcategory assignment was used as the reference standard for analyses involving BI-RADS 4 subcategories (4A, 4B, 4C). The diagnostic performance of radiologists and LLMs in distinguishing between benign and malignant lesions was assessed using the *χ*^2^ test. Cohen κ coefficient was used to evaluate pairwise agreement between each assessment method, while Fleiss κ was used to assess the consistency of the three repeated outputs from each LLM. A Fleiss κ value less than 0.2 indicated poor agreement, 0.2 to 0.4 fair agreement, 0.4 to 0.6 moderate agreement, 0.6 to 0.8 substantial agreement, and 0.8 to 1.0 almost perfect agreement. Independent samples *t* tests were used to compare the differences in lesion size between benign and malignant groups. Receiver operating characteristic curve analysis was performed to assess diagnostic performance, and the area under the receiver operating characteristic curve (AUC), sensitivity, specificity, accuracy, and *F*_1_-score were calculated. AUC values were compared using the DeLong test. A 2-sided *P* value less than .05 was considered statistically significant. All statistical analyses were performed using SPSS software (version 26.0; IBM Corp).

## Results

### Patients and Histopathological Subtypes

A total of 307 mammography reports from 307 female patients were included in this study, encompassing 309 breast lesions. The mean age of the patients was 47.25 (SD 11.39) years. Among the lesions, 152 (49.2%) were benign, and 157 (50.8%) were malignant, as determined by histopathological diagnosis. The mean size of benign lesions was 16.2 (SD 11.9) mm, and that of malignant lesions was 17.8 (SD 13.2) mm. The histopathological subtypes of the 309 BI-RADS category 4 lesions are summarized in Table 1.

**Table 1:** Histopathological findings of 309 Breast Imaging Reporting and Data System (BI-RADS) category 4 breast lesions.

Histopathological subtype			Lesions, n (%)
**Benign**			
	Fibroadenoma	48 (31.6)	
	Sclerosing lesions/adenosis	42 (27.6)	
	Intraductal papilloma	11 (7.2)	
	Inflammatory lesions	10 (6.6)	
	Cyst/hemangioma	7 (4.6)	
	Ductal ectasia	7 (4.6)	
	Benign phyllodes tumor	7 (4.6)	
	Normal breast tissue	3 (2.0)	
	Other benign findings	17 (11.2)	
	Total	152 (49.2)	
**Malignant**			
	Invasive ductal carcinoma	121 (77.1)	
	Ductal carcinoma in situ	28 (17.8)	
	Malignant phyllodes tumor	4 (2.6)	
	Invasive lobular carcinoma	2 (1.3)	
	Other malignant tumors	2 (1.3)	
	Total	157 (50.8)	

### Comparison of LLMs and Radiologists in Predicting Malignancy of BI-RADS Category 4 Lesions

If the three responses generated by an LLM were entirely inconsistent, the case was deemed diagnostically invalid. Based on this criterion, ChatGPT-4o had 3 (1.0%) invalid cases, Claude 3 Opus had 10 (3.2%), and DeepSeek-R1 had 5 (1.6%). Comparative analysis of these 18 excluded reports showed no significant differences from the included cases in report length, terminology complexity, and lesion size (all *P*>.05; Multimedia Appendix 3), indicating minimal risk of selection bias. After exclusion, the diagnostic performance of radiologists and LLMs in differentiating benign from malignant BI-RADS category 4 lesions is summarized in Table 2.

**Table 2:** Diagnostic performance of large language models and radiologists in assessing the benign or malignant nature of breast lesions.

Evaluator	Sensitivity (%; 95% CI)	Specificity (%; 95% CI)	Accuracy (%; 95% CI)	Positive predictive value (%; 95% CI)	Negative predictive value (%; 95% CI)	AUC^a^ (95% CI)	*P* value^b^
Junior radiologist	68.0 (60.2-74.9)	76.1 (68.4-82.3)	71.9 (66.5-76.8)	75.0 (67.1-81.5)	69.2 (61.6-75.9)	0.72 (0.66-0.78)	<.001
Senior radiologist	80.7 (73.6-86.2)	68.3 (60.3-75.4)	74.7 (69.4-79.3)	72.9 (69.4-79.3)	77.0 (68.9-83.5)	0.75 (0.69-0.80)	<.001
ChatGPT-4o	84.7 (78.0-89.6)	40.1 (32.4-48.4)	63.0 (57.3-68.3)	59.9 (53.2-66.3)	71.3 (60.5-80.0)	0.62 (0.56-0.69)	<.001
Claude 3 Opus	92.7 (87.3-95.9)	28.9 (22.1-36.8)	61.6 (55.9-67.0)	57.9 (51.6-64.0)	78.8 (66.0-87.8)	0.61 (0.54-0.67)	<.001
DeepSeek-R1	84.0 (77.3-89.0)	43.0 (35.1-51.2)	64.0 (58.4-69.3)	60.9 (54.1-67.3)	71.8 (61.4-80.2)	0.64 (0.57-0.70)	<.001

^a^AUC: area under the receiver operating characteristic curve.

^b^*P* values represent the test against the null hypothesis of an AUC of 0.5.

**Figure 2:**
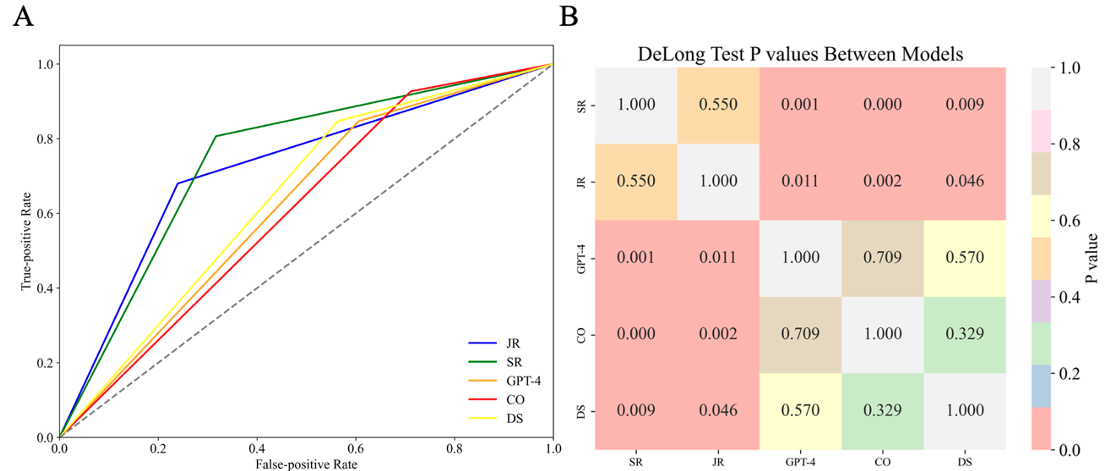
Receiver operating characteristic curve comparison of the diagnostic performance of large language models and radiologists in distinguishing between benign and malignant breast lesions. CO: Claude 3 Opus; DS: DeepSeek-R1; GPT-4: ChatGPT-4o; JR: junior radiologist; SR: senior radiologist.

DeepSeek-R1 achieved an AUC of 0.64, which was slightly higher than ChatGPT-4o (AUC 0.62) and Claude 3 Opus (AUC 0.61), but all three models underperformed compared to the junior and senior radiologists (AUC 0.72 and 0.75, respectively). The differences in AUCs between each LLM and the junior radiologist (all *P*<.05) as well as the senior radiologist (all *P*<.01) were statistically significant, indicating that both radiologists outperformed the three LLMs. In contrast, no significant differences were observed among ChatGPT-4o, Claude 3 Opus, and DeepSeek-R1, nor between the junior and senior radiologists (*P*=.55), as shown in Figure 2. The results of the DeLong tests are provided in Multimedia Appendix 4.

### Performance of LLMs in BI-RADS 4 Subcategory Assignment

Model performance for BI-RADS 4 subcategory assignment is shown in Table 3. All three LLMs demonstrated variable performance across subcategories. The 4C category consistently achieved the highest *F*_1_-scores for each model, whereas 4B showed the lowest performance overall. ChatGPT-4o and DeepSeek-R1 demonstrated comparable overall *F*_1_-scores (47.6% and 45.6%, respectively), while Claude 3 Opus showed the lowest overall performance (36.2%).

**Table 3:** Performance of large language models in Breast Imaging Reporting and Data System (BI-RADS) 4 subcategory assignment using the senior radiologist’s categorization as the reference standard.

Model and subcategories			Recall (%)		Precision (%)		*F*_1_-score (%)
**ChatGPT-4o**							
	4A	35.2		67.7		46.3	
	4B	45.1		38.5		41.6	
	4C	63.1		49.5		55.5	
	Overall	46.0		54.2		47.6	
**Claude 3 Opus**							
	4A	17.6		81.5		28.9	
	4B	23.2		31.7		26.8	
	4C	88.1		41.3		56.3	
	Overall	39.5		55.9		36.2	
**DeepSeek-R1**							
	4A	28.0		64.8		39.1	
	4B	41.5		36.6		38.9	
	4C	70.2		55.1		61.8	
	Overall	44.0		54.1		45.6	

### Consistency and Agreement Analysis of LLMs and Radiologists

Each of the three LLMs (ChatGPT-4o, Claude 3 Opus, and DeepSeek-R1) was queried three times per report to evaluate response consistency. The agreement among the three outputs was generally high across all models. As shown in Table 4, ChatGPT-4o demonstrated the highest consistency with a Fleiss κ of 0.850, followed by DeepSeek-R1 at 0.824, and Claude 3-Opus at 0.732. All three models exhibited a high level of internal consistency. The detailed frequency of BI-RADS subcategories for LLMs is presented in Multimedia Appendix 5.

To further quantify agreement across all assessment methods, a pairwise Cohen κ analysis was performed for both malignancy classification and BI-RADS 4 subcategories. The results demonstrated substantial variability in agreement among raters, with consistently higher κ values in malignancy classification (κ range 0.26-0.74) compared to subcategory assignment (κ range 0.09-0.40). Detailed statistical metrics are provided in Multimedia Appendix 6.

**Table 4:** Response consistency of large language models (LLMs) assessed by Fleiss κ statistics.

LLMs	κ value (SD)	95% CI	*z* value	*P* value
ChatGPT-4o	0.850 (0.023)	0.806-0.895	37.436	<.001
Claude 3 Opus	0.732 (0.017)	0.698-0.766	42.186	<.001
DeepSeek-R1	0.824 (0.023)	0.779-0.868	35.977	<.001

### Analysis of Diagnostic Errors by LLMs

To better understand the diagnostic behavior of the LLMs, we performed targeted misclassification analyses for DeepSeek-R1, the model with the best overall performance, and Claude 3 Opus, which showed unusually high sensitivity but low specificity.

**Figure 3:**
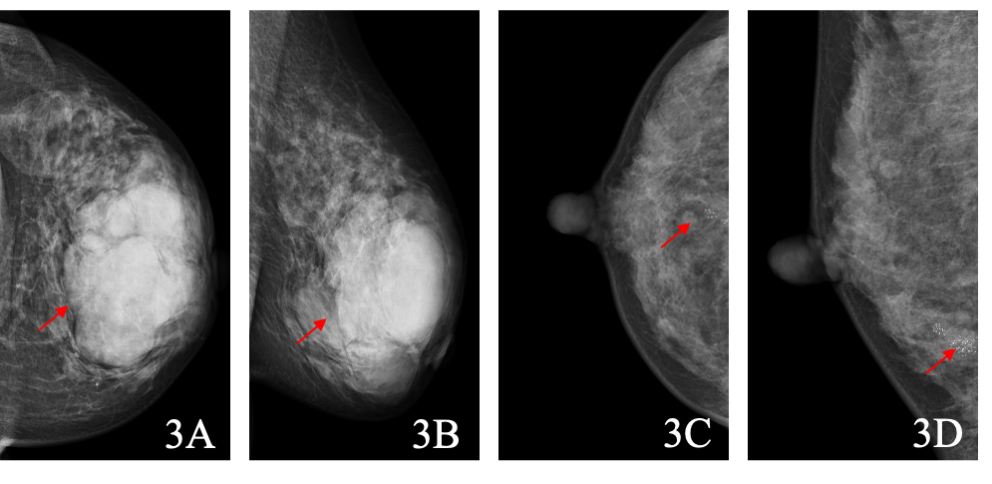
Representative mammographic cases misclassified by DeepSeek-R1. A and C are craniocaudal views; B and D are mediolateral oblique views. (A, B) A 44-year-old woman with type C breast density. A large, well-defined, shallowly lobulated mass (approximately 88 × 68 mm) is visible in the left breast. The radiologist classified the lesion as Breast Imaging Reporting and Data System (BI-RADS) 4A, while DeepSeek-R1 classified it as BI-RADS 4C. Postoperative pathology confirmed fibroadenoma. (C, D) A 37-year-old woman with type C breast density. Clustered calcifications are seen in the lower outer quadrant of the right breast, measuring approximately 16 × 11 mm. The radiologist classified the finding as BI-RADS 4A, while DeepSeek-R1 classified it as BI-RADS 3. Postoperative pathology confirmed ductal carcinoma in situ.

DeepSeek-R1 incorrectly classified 86 pathologically benign BI-RADS 4 lesions as malignant. The most common diagnoses were fibroadenoma (n=29, 33.7%), adenosis (n=20, 23.3%), intraductal papilloma (n=7, 8.1%), and inflammatory lesions (n=6, 7.0%). As shown in Table 5, lesion size was the most prominent feature cited in the reasoning text, accounting for approximately 34% of false-positive errors. Misclassified benign lesions were significantly larger than correctly classified benign lesions (mean 17.4, SD 13.9 mm vs mean 11.5, SD 8.6 mm; *P*=.01). Of the 193 extracted features, calcification descriptors constituted a significant proportion (n=49, 25.4%), encompassing both benign and suspicious subtypes. A representative example is illustrated in Figure 3A and B, where a large lobulated mass was upgraded by DeepSeek-R1 but was ultimately confirmed as fibroadenoma. In contrast, DeepSeek-R1 misclassified only 27 (8.7%) malignant cases as benign, primarily invasive ductal carcinoma (n=18, 66.7%) and ductal carcinoma in situ (n=9, 33.3%). Figure 3C and D illustrates a case classified as benign by DeepSeek-R1 but confirmed as ductal carcinoma in situ.

Claude 3 Opus produced 101 false-positive predictions, far more than DeepSeek-R1, which largely explains its markedly reduced specificity. These misclassified benign lesions included fibroadenoma (n=30, 29.7%), adenosis (n=20, 19.8%), papilloma (n=8, 7.9%), and inflammatory changes (n=7, 6.9%). Similar to DeepSeek-R1, lesion size played a major role (Table 5). Misclassified benign lesions were significantly larger than correctly classified benign lesions (mean 16.28, SD 12.64 mm vs mean 13.6, SD 8.2 mm; *P*=.01). Claude 3 Opus misclassified only 10 malignant lesions as benign (invasive ductal carcinoma: n=6), far fewer than DeepSeek-R1.

**Table 5:** Distribution of imaging descriptors identified by DeepSeek-R1 and Claude 3 Opus in the diagnostic reasoning for benign lesions misclassified as malignant.

Model and subcategory					Identified descriptors, n (%)
**DeepSeek-R1 (n=193)**					
	Size			65 (33.7)	
	**Calcifications**			49 (25.4)	
		Milk of calcium	17 (8.8)		
		Amorphous	10 (5.2)		
		Coarse	6 (3.1)		
		Calcifications	4 (2.1)		
		Round	4 (2.1)		
		Distribution	3 (1.6)		
		Fine pleomorphic	3 (1.6)		
		Large rodlike	2 (1.0)		
	Margin obscured			26 (13.5)	
	Margin microlobulated			17 (8.8)	
	Masses irregular			15 (7.8)	
	Architectural distortion			7 (3.6)	
	Margin spiculated			6 (3.1)	
	Focal asymmetry			5 (2.6)	
	Skin thickening			2 (1.0)	
	Solitary dilated duct			1 (0.5)	
**Claude 3 Opus (n=205)**					
	Size			82 (40.0)	
	**Calcification**			54 (26.3)	
		Milk of calcium	21 (10.2)		
		Fine pleomorphic	6 (2.9)		
		Distribution	5 (2.4)		
		Amorphous	4 (2.0)		
		Microcalcification	4 (2.0)		
		Calcifications	3 (1.5)		
		Nodulat	3 (1.5)		
		Coarse	2 (1.0)		
	Margin microlobulated			20 (10.0)	
	Margin obscured			13 (6.3)	
	Focal symmetry			11 (5.4)	
	Glandular aggregation			9 (4.4)	
	Margin spiculated			9 (4.4)	
	Architectural distortion			3 (1.5)	
	Masses irregular			2 (1.0)	
	High-density glands			2 (1.0)	

## Discussion

### Principal Results

LLMs have been widely recognized for their potential in medical imaging applications. However, studies evaluating the feasibility of LLM-based diagnostic reasoning against pathology-confirmed gold standards remain limited. In this study, we applied ChatGPT-4o, Claude 3 Opus, and DeepSeek-R1 to the free-text Chinese mammography reports of BI-RADS category 4 lesions, aiming to assess their capability in distinguishing between benign and malignant findings. Our results demonstrated that LLMs provide high diagnostic repeatability, good stability, and reliable outputs, supporting their feasibility in this task. Among the three models, Claude 3 Opus yielded the highest proportion of invalid predictions, while ChatGPT-4o had the lowest, suggesting that ChatGPT-4o offers greater response consistency. ChatGPT-4o also showed the highest intramodel agreement in BI-RADS classification, with minimal variation across repeated outputs. This strong consistency and stability may be attributed to the architecture of its deep learning model and the diversity of its training data, which enable ChatGPT-4o to capture subtle variations in input and deliver highly consistent judgments for the same case across different prompts. DeepSeek-R1 also demonstrated good consistency, although some fluctuations in results were observed. We speculate that this may be due to differences in model architecture and training strategies, indicating that further optimization may be needed for processing complex medical imaging reports.

At the same time, this study preliminarily explored the feasibility of using three LLMs, ChatGPT-4o, Claude 3 Opus, and DeepSeek-R1 to accurately assess the malignancy of BI-RADS category 4 lesions based on free-text Chinese mammography reports, using pathological results as the reference standard. The findings showed that all three models were capable of predicting benign versus malignant outcomes for BI-RADS 4 lesions with high sensitivity: 84.7% for ChatGPT-4o, 92.7% for Claude 3 Opus, and 84.0% for DeepSeek-R1. These values were higher than those of junior (68.0%) and senior (80.7%) radiologists. Sensitivity is considered a critical metric for ensuring early detection of malignancies, particularly in the initial screening phase, where reducing missed diagnoses is essential. Consistent with our results, Pagano et al [34] evaluated five different LLMs and compared their sensitivity in diagnosing hip and knee osteoarthritis. Their findings showed that GPT-4o outperformed the other models, achieving a sensitivity of 92.3%. Similarly, Laohawetwanit et al [35] used GPT-4 to classify histopathological images of colorectal adenomas and reported a high sensitivity of 74% for adenoma detection. In the field of breast imaging, Miaojiao et al [36] evaluated ChatGPT-4 for BI-RADS classification of breast ultrasound nodules and reported a sensitivity of 90.56% in distinguishing malignant lesions, further demonstrating the model’s capability in cancer-related diagnostic tasks. In our study, the sensitivity of LLMs for distinguishing benign from malignant BI-RADS 4 lesions reached up to 92.7%, further supporting the potential of LLMs in breast cancer screening. These findings suggest that LLMs could serve as effective high-sensitivity decision support tools to help radiologists, particularly junior physicians, rapidly flag and prioritize high-risk cases in routine breast cancer screening workflows.

This study demonstrates that LLMs exhibit the characteristic of high sensitivity but relatively low specificity in interpreting imaging reports. Previous studies have reported similar findings. Sievert et al [23] showed that, in thyroid nodule risk stratification, ChatGPT achieved a sensitivity of 86.7% to 94.1%, whereas specificity was only 10.7% to 18.2%. Shi et al [37] used GPT-4 to automatically generate biopsy recommendations by integrating prostate reports with clinical data; their results revealed that GPT-4 achieved a sensitivity of 0.84 to 0.90 but a specificity of 0.41 to 0.44 for prostate biopsy triage, indicating an advantage in reducing missed diagnoses but a limitation as a stand-alone diagnostic tool. Yang et al [38] evaluated the ability of ChatGPT to differentiate benign from malignant bone tumors using 1366 imaging reports and further optimized performance through few-shot prompting. In the baseline setting, the model achieved a sensitivity of 0.95 and a specificity of 0.58, again suggesting potential to reduce missed diagnoses but with insufficient specificity. The principal reasons for the low specificity of LLMs may include an overreliance on malignant lexical cues and surface features. When reports contain descriptors such as “microcalcification” or “ill-defined margin,” the model tends to upgrade the risk level while inadequately integrating benign evidence. In addition, LLMs possess limited capability to integrate contextual information, especially when only text is provided without imaging, resulting in a lack of multimodal information to support comprehensive judgment [39]. These mechanisms indicate that the optimal clinical role of LLMs is as a screening or triage tool; they can rapidly flag high-risk cases, standardize key findings, and assist radiologists in decision-making, thereby reducing missed diagnoses.

Regrettably, our findings indicate that although DeepSeek-R1, ChatGPT-4o, and Claude 3 Opus are capable of performing benign versus malignant classification for BI-RADS category 4 lesions based on free-text reports, their overall diagnostic performance remains inferior to that of radiologists when benchmarked against pathological results. This observation is consistent with recent literature. Wu et al [26] compared GPT-4, GPT-3.5, and Google Bard in predicting the malignancy of thyroid nodules using pathology as the reference standard. They found that all three LLMs achieved diagnostic performance above 0.8, outperforming junior physicians but still falling short of senior radiologists. Similarly, Liu et al [40] evaluated GPT-4.0 and Bing using ultrasound-based breast imaging reports and demonstrated that GPT-4 outperformed Bing, yet remained inferior to human radiologists. These results suggest that, despite significant advancements, LLMs have not yet fully matched human expertise in medical imaging interpretation. Notably, the diagnostic performance of the LLMs in our study was even lower than that reported by Liu et al [40], which may be due to differences in study populations. While Liu et al [40] included lesions across the full BI-RADS spectrum (categories 2 through 5), our study focused exclusively on BI-RADS category 4, a subset known for its diagnostic ambiguity and overlapping imaging features between benign and malignant lesions. Supporting this, a study by Elezaby et al [41] analyzed data from the US National Mammography Database between 2008 and 2014, covering 125,447 BI-RADS 4 cases [41]. Among them, 33.3% were subcategorized into 4A, 4B, and 4C, with corresponding positive predictive values of 7.6%, 22.2%, and 69.3%, respectively. This large-scale dataset highlights the intrinsic challenge of accurately characterizing BI-RADS 4 lesions, even for experienced radiologists, and further underscores the difficulty faced by LLMs when attempting to replicate expert-level discrimination within this category.

Interestingly, no significant difference in BI-RADS diagnostic performance was observed between junior and senior radiologists (*P*=.55), which may be attributable to several factors. First, the BI-RADS system provides highly standardized terminology and structured reporting, offering clear imaging feature descriptions and well-defined categorization rules. This markedly reduces interobserver variability and enables radiologists with different levels of experience to reach comparable diagnostic conclusions. Cozzi et al [28] demonstrated in a multilingual study that even when interpretation was based solely on the “imaging findings” section of reports, interreader agreement remained nearly perfect (Gwet AC1 0.91), underscoring the strong harmonizing effect of the BI-RADS framework on experience-related differences. Second, the sample size and lesion composition of the present study may have influenced the results. The enrolled cases displayed a relatively balanced benign-to-malignant ratio but lacked a large number of highly challenging or borderline lesions, which may have limited the opportunity for differences in reader experience to emerge. Finally, the evaluation criteria themselves, final BI-RADS categories confirmed by pathology or follow-up, with diagnostic performance assessed by DeLong testing, focused on objective diagnostic accuracy rather than subtle variations in descriptive detail. This emphasis on definitive outcomes may have further attenuated measurable differences between radiologists of different seniority.

The relatively low specificity and diagnostic performance of LLMs compared to radiologists have not been thoroughly investigated in previous literature [42]. In this study, using pathology as the reference standard, we attempted to analyze the internal reasoning behind LLM outputs. The diagnostic outcomes of LLMs are influenced by various factors, including the weight distribution of their training datasets and the content of the prompts used. In our case, we used the default versions of ChatGPT-4o, Claude 3 Opus, and DeepSeek-R1 without any additional fine-tuning on domain-specific medical content, which may have contributed to some diagnostic inaccuracies. Regarding prompt design, LLMs primarily rely on extracting and interpreting keywords from BI-RADS mammography reports, such as lesion size, morphology, margins, and calcifications, to make diagnostic decisions. Among these, lesion size emerged as a dominant factor. We observed that lesions with a short-axis diameter greater than 17 mm were often classified as BI-RADS 4B or higher by the models, suggesting a malignant tendency. This aligns with clinical practice, where lesion size is also a critical consideration for radiologists. Our findings are consistent with those of Ong et al [43], who reported that lesion size greater than or equal to 15 mm was an independent predictor of malignancy in contrast-enhanced mammography (adjusted odds ratio 22.5), significantly increasing the likelihood of a malignant diagnosis. Furthermore, prior studies have shown that when the lesion size reaches 17.5 mm, specificity improves to 89.7%, reinforcing the value of size as a key imaging-based risk stratification parameter. Therefore, when using LLMs to assist in further benign versus malignant differentiation of BI-RADS category 4 lesions, caution is warranted for large lesions, as LLMs are more likely to overpredict malignancy. Our data showed a misclassification rate of approximately 34% for benign lesions larger than 17 mm. In addition to lesion size, the handling of the coexistence of benign and suspicious imaging descriptors was also a key reason for false positives. Although LLMs explicitly mentioned typically benign features such as “milk of calcium” during the reasoning process, they struggled to appropriately weigh them against co-occurring, seemingly suspicious features (such as large lesion size, high density, or asymmetry). In a clinical setting, a definitive benign feature should typically be sufficient to de-escalate the risk classification. However, the LLMs failed to assign sufficient weight to this benign evidence to override other findings. Instead, the LLMs appeared to allow the co-occurring suspicious features to dominate the decision-making process, thereby leading to false-positive malignant diagnoses (BI-RADS 4B or 4C). Fibroadenoma and adenosis were the two most common benign lesions misclassified by LLMs, accounting for 34% and 21% of misdiagnosed benign cases, respectively. These lesions were often relatively large, with lobulated or indistinct margins, and sometimes associated with calcifications. As such, these features likely prompted the LLMs to overpredict malignancy. This suggests that one of the main limitations affecting the diagnostic performance of LLMs lies in their tendency to overrely on certain high-risk imaging features. In summary, we believe that the principal limitation of LLMs lies in keyword-triggered escalation. When a mammography report contains malignant-leaning cues, such as spiculated margins, pleomorphic or clustered calcifications, architectural distortion, or indistinct or lobulated borders, or describes a relatively large lesion, the models tend to assign higher BI-RADS categories (4B or 4C). This behavior reflects an overreliance on surface lexical cues and insufficient integration of benign evidence. To mitigate this limitation, future work should refine prompt design to require balanced and explicit extraction of all malignant and benign descriptors, and to discourage upgrades based on any single malignant cue or lesion size alone, which may guide LLMs toward more comprehensive reasoning.

### Limitations

This study has several limitations. First, this is a single-center, retrospective analysis based on free-text mammography reports, which may limit the generalizability of the findings and introduce potential selection bias. Future studies should incorporate a more diverse, multicenter dataset with prospective validation to improve external validity. Second, the results are highly dependent on the design of the prompts used to query the LLMs. Only one optimized prompt was applied in this study, and no systematic comparison of different prompt structures was performed. Future research should include multiple prompt designs and iterative optimization, including different language prompts to assess and enhance the stability and reproducibility of model performance. Third, the LLMs used in this study were not fine-tuned on domain-specific medical data and were evaluated in their default general-purpose form. This lack of domain adaptation may limit their ability to fully capture breast imaging and specific diagnostic patterns and to integrate nuanced clinical knowledge. Future work could include domain-specific fine-tuning, for example, training on large curated radiology report datasets or incorporating expert-reviewed guidelines, to further optimize diagnostic reasoning and improve model performance. Fourth, cases in which the LLMs failed to reach a majority consensus were excluded from analysis. This may introduce selection bias and could result in an overestimation of consistency and diagnostic performance, as the reported metrics reflect only those cases in which the model outputs were sufficiently stable.

### Conclusions

In conclusion, this study demonstrated that all three LLMs, ChatGPT-4o, Claude 3 Opus, and DeepSeek-R1 have high sensitivity in predicting BI-RADS category 4 breast benign and malignant lesions with good repeatability and stability but relatively insufficient specificity. The main causes of misclassification by LLMs included larger lesion size (short axis >17 mm) and the presence of specific imaging features described in the reports, such as clustered calcifications, spiculated margins, lobulated contours, and indistinct edges. Fibroadenoma and adenosis were the most common benign lesions misclassified as malignant by the LLMs.

## Appendix

Multimedia Appendix 1 Chinese prompt and example outputs from three large language models.

Multimedia Appendix 2 Prompt versions tested during pilot runs and associated output issues.

Multimedia Appendix 3 Comparison of characteristics between excluded and included cases.

Multimedia Appendix 4 DeLong test results for large language models' area under the receiver operating characteristic curve comparisons.

Multimedia Appendix 5 Classification distributions of BI-RADS subcategories for LLMs.

Multimedia Appendix 6 Pairwise Cohen κ analysis for malignancy classification and Breast Imaging Reporting and Data System (BI-RADS) 4 subcategories.

## Abbreviations

AI: artificial intelligence
AUC: area under the receiver operating characteristic curve
BI-RADS: Breast Imaging Reporting and Data System
GPT: generative pretrained transformer
LLM: large language model
