# Supplementary figures and images for: Performance of ChatGPT-4o, Claude 3 Opus, and DeepSeek-R1 in BI-RADS Category 4 Classification and Malignancy Prediction From Mammography Reports: Retrospective Diagnostic Study
Source: JMIR Med Inform. 2025 Dec 25;13:e80182. doi: 10.2196/80182 (PMC12784141; doi:10.2196/80182)

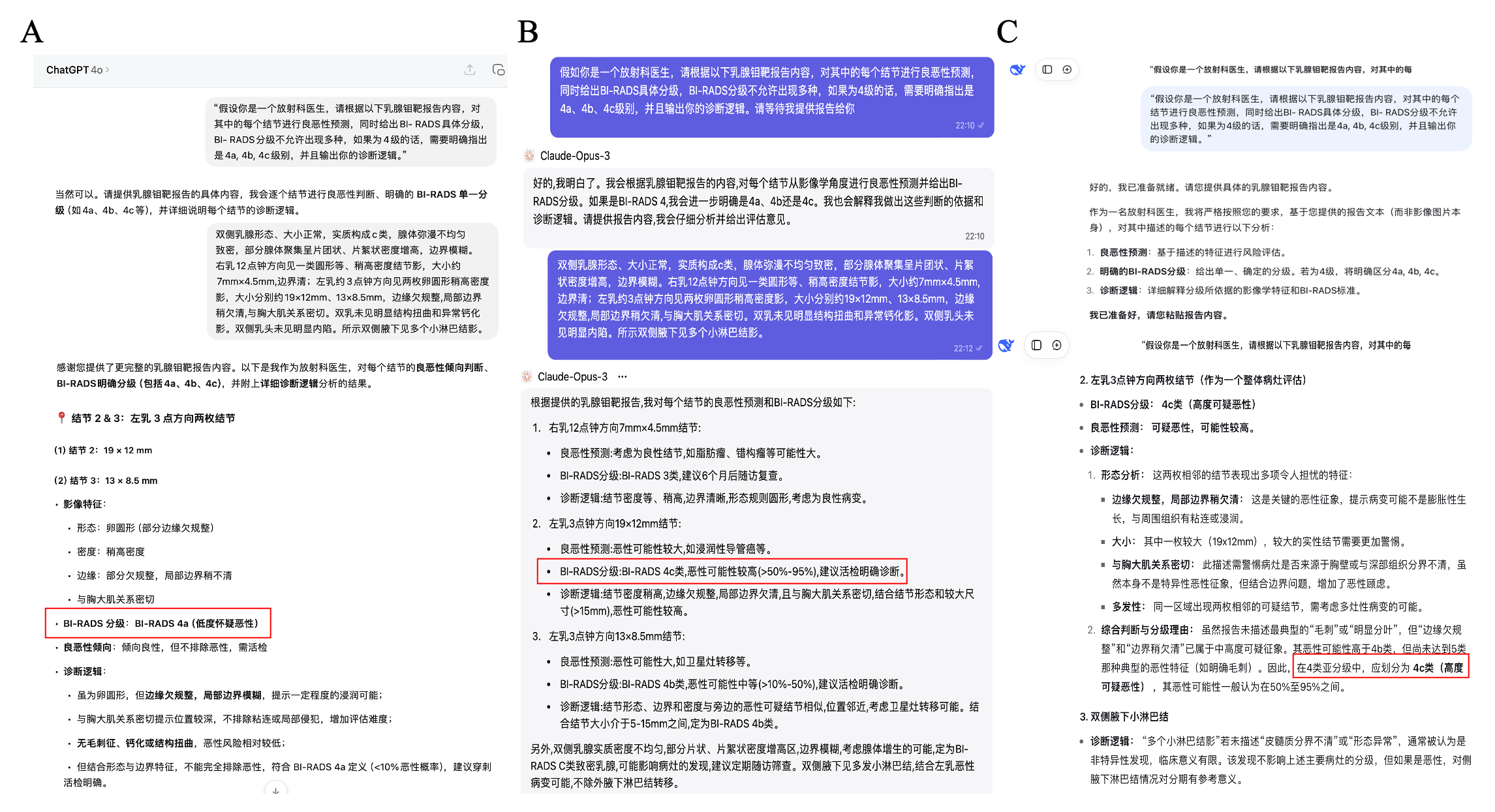

Supplement: Multimedia Appendix 1 [file medinform_v13i1e80182_app1.png]
