# Supplementary material for: Performance of ChatGPT-4o, Claude 3 Opus, and DeepSeek-R1 in BI-RADS Category 4 Classification and Malignancy Prediction From Mammography Reports: Retrospective Diagnostic Study
Source: JMIR Med Inform. 2025 Dec 25;13:e80182. doi: 10.2196/80182 (PMC12784141; doi:10.2196/80182)
Supplement: Multimedia Appendix 2 [file medinform_v13i1e80182_app2.docx]

Multimedia Appendix 2

Prompt Versions Tested During Pilot Runs and Associated Output Issues

| Prompt Version | Prompt | Observed Output Behavior | Limitations |
| --- | --- | --- | --- |
| Initial  (Version 1) | Please read the mammography report, give a BI-RADS grade for each nodule, and determine whether it is benign or malignant. | (a) Output included BI-RADS categories like “4B or 4C”  (b) Often lacked explanation for classification  (c) Sometimes gave multiple BI-RADS levels per lesion | (a) Multiple classifications  (b) Lack of diagnostic reasoning  (c) Ambiguous assignments |
| Optimized  (Version 2) | Please read the following mammography report, determine whether the nodule is benign or malignant, and give a BI-RADS classification (if it is category 4, please indicate 4A, 4B or 4C), and explain the reasons. | (a) BI-RADS category was more specific  (b) Some responses still included dual options (e.g., “4B/4C”)  (c) Reasoning was occasionally superficial or missing | (a) Improved structure, but not strict enough  (b) Continued ambiguity in output  (c) Inconsistent explanation quality |
| Final  (Version 3) | Assume you are a radiologist. Based on the following mammography report, please predict the benign or malignant nature of each nodule and provide a specific BI-RADS classification. Only one BI-RADS category is allowed. If classified as category 4, please clearly specify whether it is 4A, 4B, or 4C, and explain your diagnostic reasoning. | (a) Gave exactly one BI-RADS level per lesion  (b) Subcategories (4A/4B/4C) clearly specified  (c) Reasoning included in nearly all outputs | (a) No major issues observed  (b) Output structure stable and interpretable  (c) Selected as final prompt for study |
