# Supplementary material for: Performance of ChatGPT-4o, Claude 3 Opus, and DeepSeek-R1 in BI-RADS Category 4 Classification and Malignancy Prediction From Mammography Reports: Retrospective Diagnostic Study
Source: JMIR Med Inform. 2025 Dec 25;13:e80182. doi: 10.2196/80182 (PMC12784141; doi:10.2196/80182)
Supplement: Multimedia Appendix 3 [file medinform_v13i1e80182_app3.docx]

Multimedia Appendix 3

Comparison of Characteristics Between Excluded and Included Cases

| Characteristic | Excluded (n=18) | Included (n=289) | *P* |
| --- | --- | --- | --- |
| Report length (words count) | 142.4±29.8 | 149.6±30.1 | .327 |
| Terminology complexity (%) | 2.73% | 2.83% | .480 |
| Lesion size (mm) | 18.0±11.9 | 16.9±8.9 | .742 |

Terminology complexity: the proportion of words or terms not present in a predefined mammography-specific lexicon derived from RadLex.
