# Supplementary material for: Performance of ChatGPT-4o, Claude 3 Opus, and DeepSeek-R1 in BI-RADS Category 4 Classification and Malignancy Prediction From Mammography Reports: Retrospective Diagnostic Study
Source: JMIR Med Inform. 2025 Dec 25;13:e80182. doi: 10.2196/80182 (PMC12784141; doi:10.2196/80182)
Supplement: Multimedia Appendix 4 [file medinform_v13i1e80182_app4.docx]

Multimedia Appendix 4

DeLong Test Results for LLMs’ AUC Comparisons

| Model 1 | Model 2 | AUC difference | Standard error | Z statistic | *P* |
| --- | --- | --- | --- | --- | --- |
| SR | JR | 0.025 | 0.041 | 0.597 | .550 |
| SR | GPT-4 | 0.124 | 0.039 | 3.189 | .001 |
| SR | CO | 0.137 | 0.037 | 3.739 | <.001 |
| SR | DS | 0.103 | 0.039 | 2.620 | .009 |
| JR | GPT-4 | 0.100 | 0.039 | 2.548 | .011 |
| JR | CO | 0.113 | 0.037 | 3.055 | .002 |
| JR | DS | 0.079 | 0.040 | 1.988 | .046 |
| GPT-4 | CO | 0.013 | 0.034 | 0.373 | .709 |
| GPT-4 | DS | -0.021 | 0.037 | -0.567 | .570 |
| CO | DS | -0.034 | 0.035 | -0.975 | .329 |

SR: senior radiologist; JR: junior radiologist; GPT-4: ChatGPT-4o; CO: Claude 3-Opus; DS: DeepSeek
