# Supplementary material for: Performance of ChatGPT-4o, Claude 3 Opus, and DeepSeek-R1 in BI-RADS Category 4 Classification and Malignancy Prediction From Mammography Reports: Retrospective Diagnostic Study
Source: JMIR Med Inform. 2025 Dec 25;13:e80182. doi: 10.2196/80182 (PMC12784141; doi:10.2196/80182)
Supplement: Multimedia Appendix 5 [file medinform_v13i1e80182_app5.docx]

Multimedia Appendix 5

Detailed Frequency of BI-RADS Subcategories (4A, 4B, 4C) for LLMs

| Pathology | ChatGPT-4o | | | | | Claude 3-Opus | | | | | DeepSeek-R1 | | | | |
| --- | --- | --- | --- | --- | --- | --- | --- | --- | --- | --- | --- | --- | --- | --- | --- |
|  | 2-3 | 4A | 4B | 4C | 5 | 2-3 | 4A | 4B | 4C | 5 | 2-3 | 4A | 4B | 4C | 5 |
| Benign | 13 | 43 | 50 | 35 | 1 | 17 | 24 | 34 | 67 | 0 | 24 | 38 | 51 | 28 | 1 |
| Malignant | 0 | 23 | 46 | 72 | 9 | 7 | 4 | 26 | 112 | 1 | 7 | 16 | 42 | 79 | 6 |
