# Supplementary material for: Performance of ChatGPT-4o, Claude 3 Opus, and DeepSeek-R1 in BI-RADS Category 4 Classification and Malignancy Prediction From Mammography Reports: Retrospective Diagnostic Study
Source: JMIR Med Inform. 2025 Dec 25;13:e80182. doi: 10.2196/80182 (PMC12784141; doi:10.2196/80182)
Supplement: Multimedia Appendix 6 [file medinform_v13i1e80182_app6.docx]

Multimedia Appendix 6

**Pairwise Cohen’s Kappa Analysis for Malignancy Classification and BI-RADS 4 Subcategories**

| Task | Comparison | Kappa Value | z Value | *P* | 95% CI |
| --- | --- | --- | --- | --- | --- |
| Malignancy | JR vs SR | 0.734 | 18.307 | <.01 | 0.656–0.813 |
|  | JR vs GPT-4 | 0.265 | 4.761 | <.01 | 0.156–0.374 |
|  | JR vs CO | 0.201 | 3.605 | <.01 | 0.092–0.310 |
|  | JR vs DS | 0.331 | 6.049 | <.01 | 0.224–0.438 |
|  | SR vs GPT-4 | 0.346 | 5.978 | <.01 | 0.232–0.459 |
|  | SR vs CO | 0.289 | 4.787 | <.01 | 0.171–0.407 |
|  | SR vs DS | 0.393 | 6.989 | <.01 | 0.283–0.504 |
|  | GPT-4 vs CO | 0.459 | 6.986 | <.01 | 0.330–0.588 |
|  | GPT-4 vs DS | 0.555 | 9.942 | <.01 | 0.446–0.665 |
|  | CO vs DS | 0.46 | 7.172 | <.01 | 0.334–0.586 |
| Subcategories | JR vs SR | 0.695 | 17.429 | <.01 | 0.617–0.773 |
|  | JR vs GPT-4 | 0.18 | 3.805 | <.01 | 0.087–0.273 |
|  | JR vs CO | 0.089 | 2.033 | <.05 | 0.003–0.174 |
|  | JR vs DS | 0.207 | 4.24 | <.01 | 0.112–0.303 |
|  | SR vs GPT-4 | 0.263 | 5.834 | <.01 | 0.174–0.351 |
|  | SR vs CO | 0.17 | 3.839 | <.01 | 0.083–0.257 |
|  | SR vs DS | 0.265 | 5.715 | <.01 | 0.174–0.357 |
|  | GPT-4 vs CO | 0.126 | 2.439 | <.05 | 0.025–0.227 |
|  | GPT-4 vs DS | 0.293 | 5.945 | <.01 | 0.197–0.390 |
|  | CO vs DS | 0.26 | 4.863 | <.01 | 0.155–0.365 |

Task includes malignancy classification (2/3/4A = benign; 4B/4C/5 = malignant) and BI-RADS 4 subcategories (4A/4B/4C only). JR = junior radiologist; SR = senior radiologist, CO = Claude 3-Opus, DS = DeepSeek.
